# Supplementary material for: Remodeling of mRNA by eIF4F in human translation initiation
Source: bioRxiv. 2026 Jun 19:2026.06.19.733470. Preprint. [Version 1] doi: 10.64898/2026.06.19.733470 (PMC13308167; doi:10.64898/2026.06.19.733470)
Supplement: 2 [file NIHPP2026.06.19.733470v1-supplement-2.pdf]

bioRxiv preprint doi: https://doi.org/10.64898/2026.06.19.733470; this version posted June 19, 2026. The copyright holder for this preprint (which was not certified by peer review) is the author/funder, who has granted bioRxiv a license to display the preprint in perpetuity. It is made available under a CC-BY 4.0 International license.

Supplementary Figures

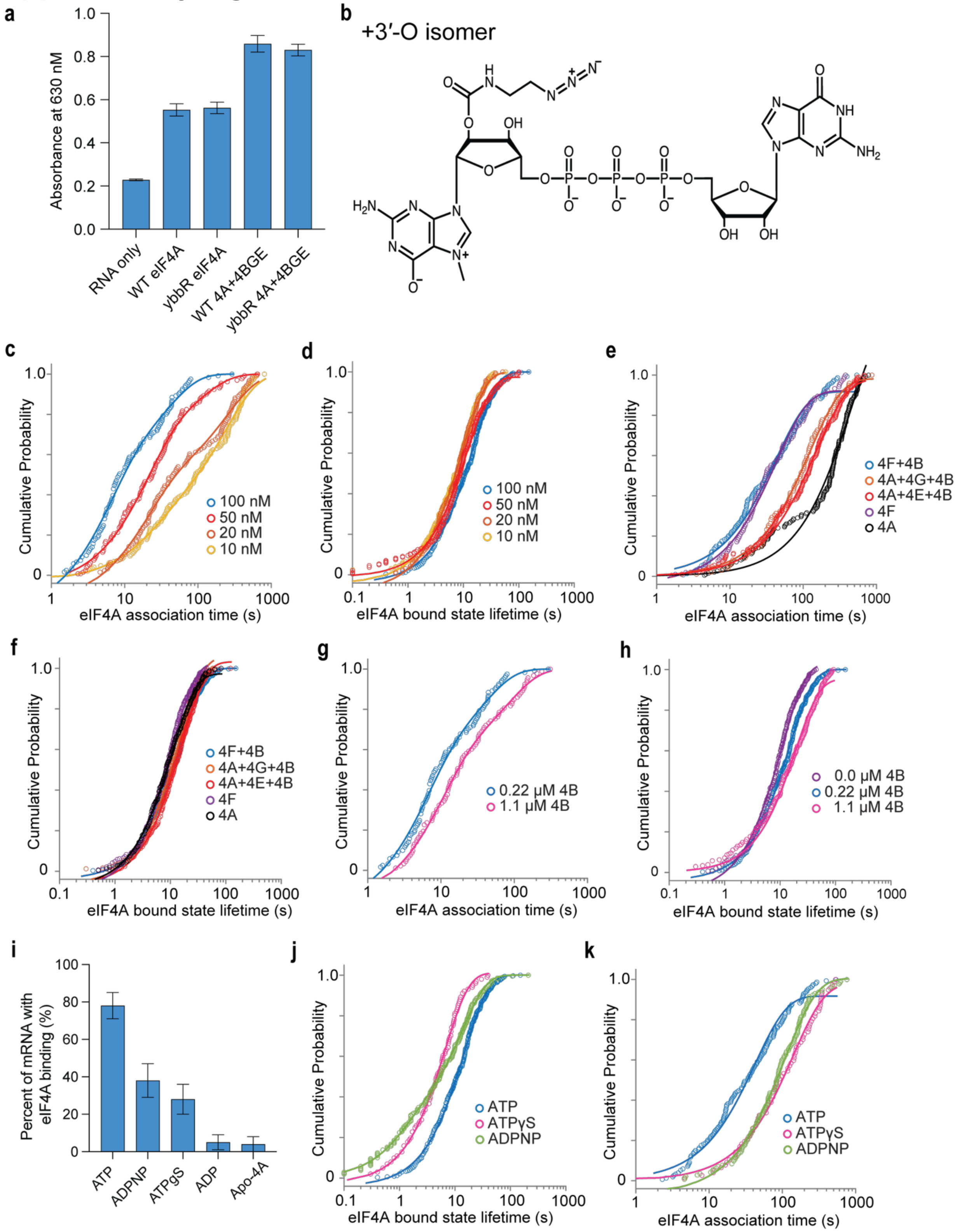

Figure S1. A 5′ end-to-eIF4A FRET assay

bioRxiv preprint doi: https://doi.org/10.64898/2026.06.19.733470; this version posted June 19, 2026. The copyright holder for this preprint (which was not certified by peer review) is the author/funder, who has granted bioRxiv a license to display the preprint in perpetuity. It is made available under a CC-BY 4.0 International license.

a. Bar plot of absorbance at 630 nm for the indicated conditions from a malachite green ATPase assay. Error bars represent standard deviation of data points, n = 2 for all conditions. **b.** Chemical structure of the cap analog (N_3-m^7GpppG) used for click chemistry labeling of capped mRNA. **c-h.** Cumulative probability plots of the indicated parameters at differing eIF4F concentrations reported by eIF4A concentration (**c** and **d**), differing eIF4 B, G, E dropouts (**e** and **f**), or differing 4B concentrations (**g** and **h**). Lines represent fits to exponential functions. Sample size is reported in Figures 1d, e. **i.** Bar plot of percent of mRNA molecules with Cy5-eIF4A binding events in differing ATP analog conditions. Error bars represent 95% CI from binomial bootstrapping. From left to right n = 111, 107, 121, 132, and 112. **j, k.** Cumulative probability plots of the indicated parameters with differing ATP analogs. Lines represent fits to exponential functions, sample size is reported in Figure 1d.

bioRxiv preprint doi: https://doi.org/10.64898/2026.06.19.733470; this version posted June 19, 2026. The copyright holder for this preprint (which was not certified by peer review) is the author/funder, who has granted bioRxiv a license to display the preprint in perpetuity. It is made available under a CC-BY 4.0 International license.

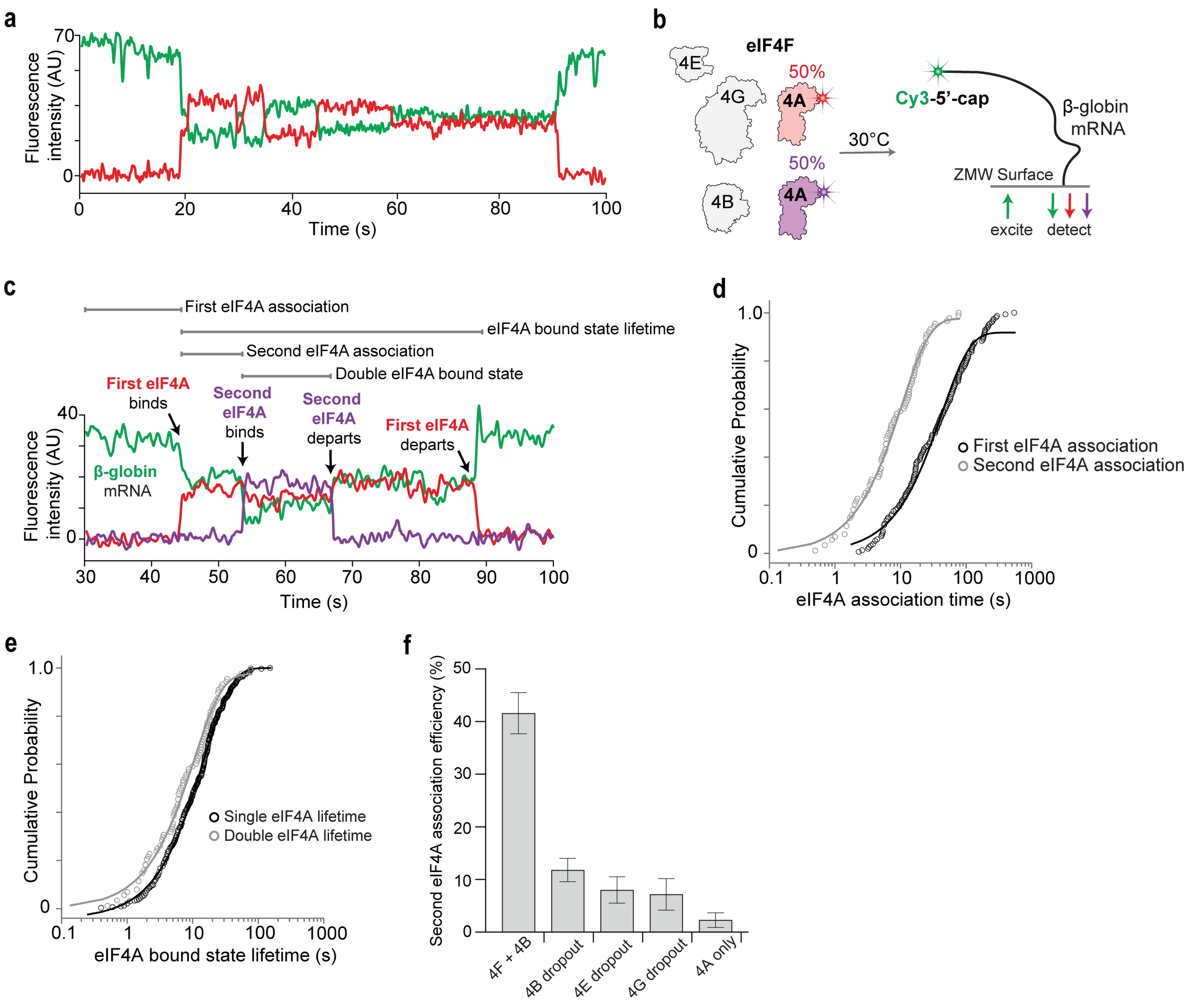

Figure S2. Multiple eIF4A bind proximal to the 5′ cap

a. Example single-molecule data showcasing multiple FRET states observed in the assay shown from Figure 1a. **b.** Schematic of single-molecule cap-to-eIF4A FRET assay where Cy5-labeled and Cy5.5-labeled eIF4A alongside eIF4E, eIF4G, eIF4B, and ATP are delivered to Cy3-5′-cap labeled β-globin mRNA immobilized on a ZMW imaging surface. **c.** Example single-molecule trace for the assay in (**b**), bursts in red or purple intensity anti-correlated with green intensity decreases (Cy3-labeled β-globin) indicate labeled eIF4A binding. The association times and lifetimes of multiple distinct eIF4A can be tracked. **d, e.** Cumulative probability plots of the indicated parameters, comparing first and second eIF4A binding events. The lines represent fits to exponential functions, for first binding n = 115, and for second binding n = 84. **f.** Bar plot for the fraction of mRNA with double eIF4A binding on the indicated conditions. From left to right n = 323, 177, 199, 201, and 210.

bioRxiv preprint doi: https://doi.org/10.64898/2026.06.19.733470; this version posted June 19, 2026. The copyright holder for this preprint (which was not certified by peer review) is the author/funder, who has granted bioRxiv a license to display the preprint in perpetuity. It is made available under a CC-BY 4.0 International license.

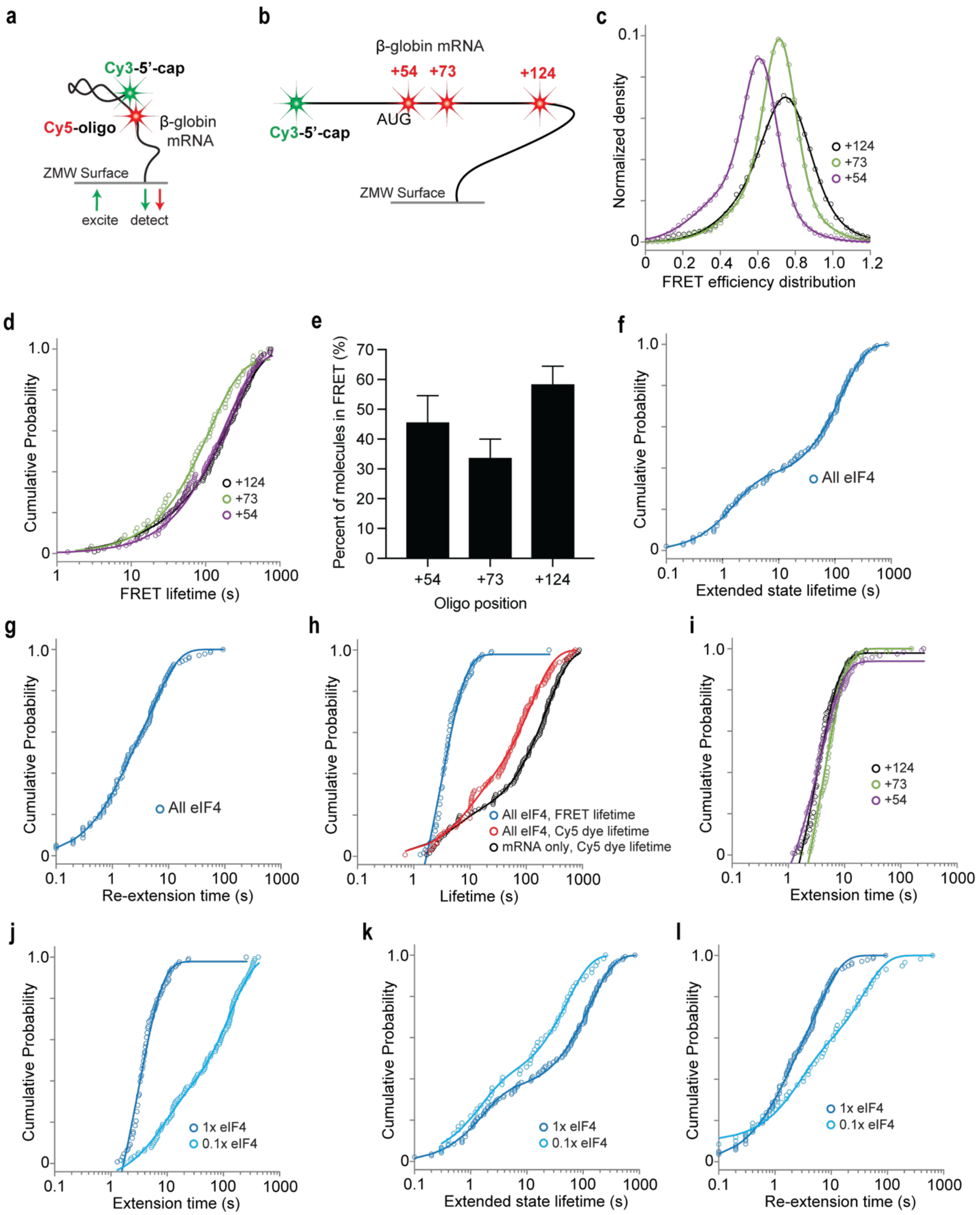

Figure S3. A cap-to-oligo FRET assay

a. Schematic of single-molecule assay to probe conformation at the 5′ end of β-globin mRNA. A Cy3-5′-cap labeled β-globin mRNA is hybridized to a Cy5-probe and immobilized on a ZMW imaging surface. **b.** Schematic showcasing the different Cy5-

bioRxiv preprint doi: https://doi.org/10.64898/2026.06.19.733470; this version posted June 19, 2026. The copyright holder for this preprint (which was not certified by peer review) is the author/funder, who has granted bioRxiv a license to display the preprint in perpetuity. It is made available under a CC-BY 4.0 International license.

probe positions used relative to the 5′ cap. **c.** Plots of FRET efficiency distribution for the different probe positions. Lines indicate fits to Gaussian functions (Values for each variable in the fit displayed in Table S4). For +54, +73, and +124: n = 124, 56, and 121. **d.** Cumulative probability plots of the FRET lifetime for the indicated probe positions. Lines represent fits to exponential functions. **e.** Plots of percent of molecules in FRET at each probe position indicated. Error bars represent 95% CI from binomial bootstrapping. From left to right, n = 108, 200, 225. **f, g.** Cumulative probability plots of the indicated parameters with all eIF4 protein in cap-to-probe FRET assay using probe position +124. The lines represent fits to exponential functions. **h.** Cumulative probability plot of lifetimes for the indicated conditions. Lines represent fits to exponential functions. **i.** Cumulative probability plots of extension time comparing different Cy5-probe positions. Lines represent fits to exponential functions. For +54, +73, +124: n = 118, 130, and 131. **j-l.** Cumulative probability plots of the indicated parameters, at two different eIF4 protein concentrations. The lines represent fits to exponential functions, for 0.1x eIF4 n = 116 molecules were analyzed.

bioRxiv preprint doi: https://doi.org/10.64898/2026.06.19.733470; this version posted June 19, 2026. The copyright holder for this preprint (which was not certified by peer review) is the author/funder, who has granted bioRxiv a license to display the preprint in perpetuity. It is made available under a CC-BY 4.0 International license.

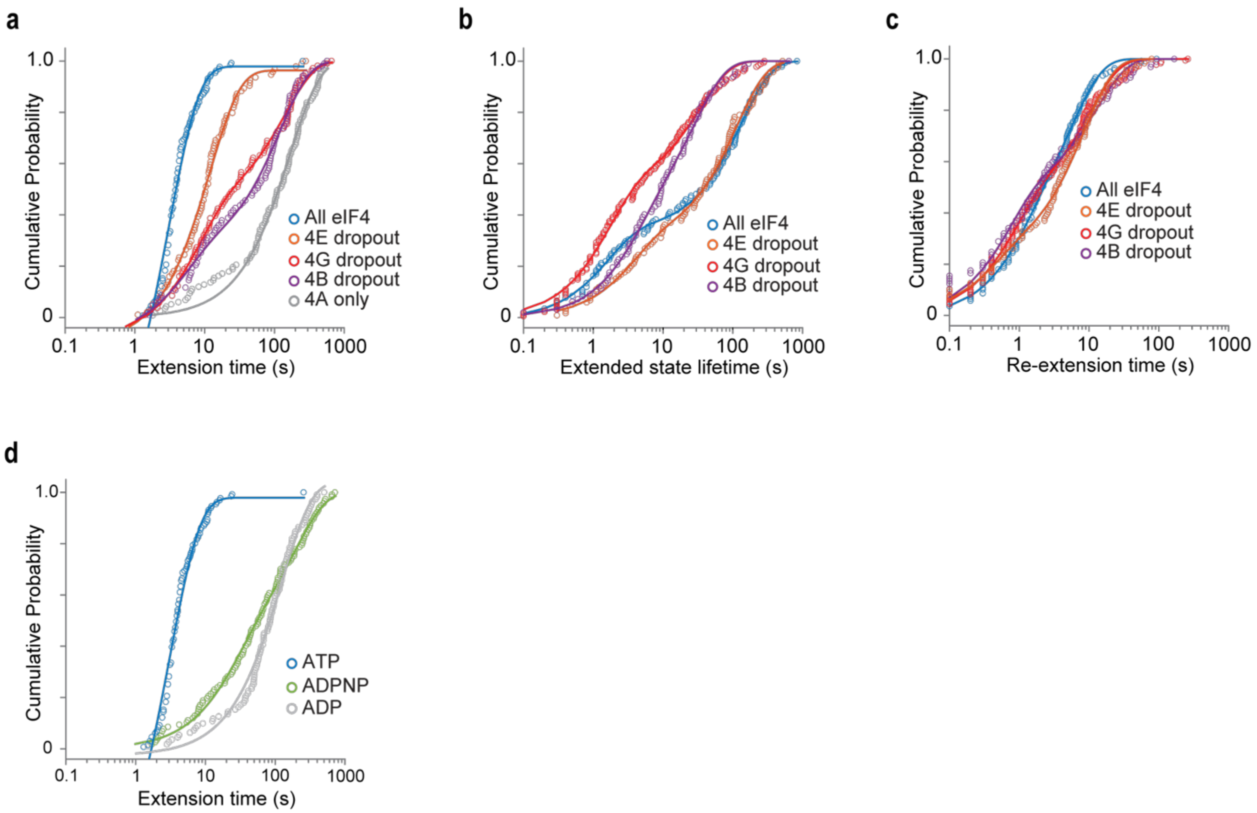

Figure S4. Factor omissions and ATP modulate mRNA extension
a-d. Cumulative probability plots of the indicated parameters for either various eIF4B, 4G, or 4E dropouts (**a**-**c**) or different ATP analogs (**d**). The lines represent fits to exponential functions; sample size is detailed in Figure 3d.

bioRxiv preprint doi: https://doi.org/10.64898/2026.06.19.733470; this version posted June 19, 2026. The copyright holder for this preprint (which was not certified by peer review) is the author/funder, who has granted bioRxiv a license to display the preprint in perpetuity. It is made available under a CC-BY 4.0 International license.

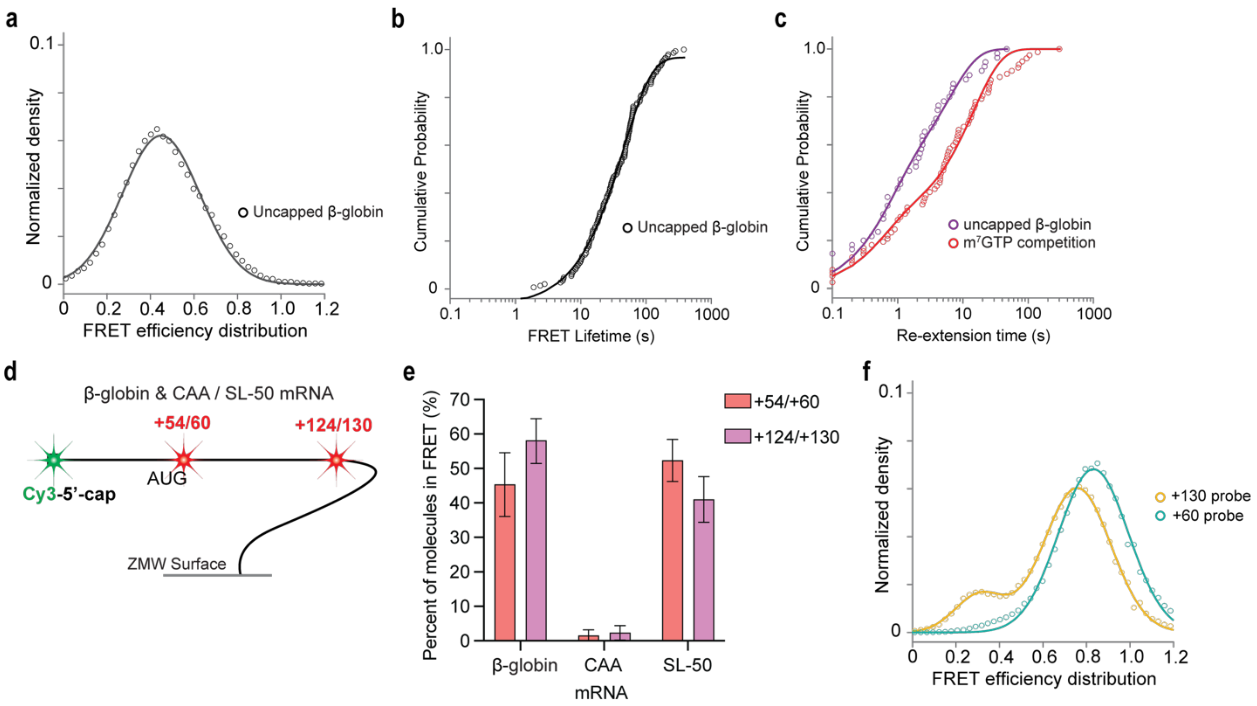

Figure S5. Characterizing model mRNA constructs in cap-to-oligo FRET assay
a, b. Plot of FRET efficiency distribution (**a**) and cumulative probability plot (**b**) for Cy3-labeled uncapped β-globin mRNA to Cy5-probe annealed at position +124. Line represents Gaussian function fit (**a**) (see Table S4 for values of the fit) or exponential function fit (**b**), n = 147. **c.** Cumulative probability plots of subsequent FRET lifetimes for the indicated conditions. Lines represent fits to exponential functions. **d.** Schematic showcasing the different Cy5-probe positions used relative to the 5′ cap with positions given for β-globin or CAA mRNA (+54 and +124) and SL-50 mRNA (+60 and +130). **e.** Bar plots of percent of molecules in FRET for the indicated mRNA at two different probe positions as shown in (**d**). Error bars represent 95% CI from binomial bootstrapping, from left to right n = 108, 225, 250, 251, 250, and 248. **f.** Plot of FRET efficiency distribution for Cy3-labeled SL-50 mRNA to Cy5-probe annealed at indicated positions. Sample size is reported in Figures 4e, f.

bioRxiv preprint doi: https://doi.org/10.64898/2026.06.19.733470; this version posted June 19, 2026. The copyright holder for this preprint (which was not certified by peer review) is the author/funder, who has granted bioRxiv a license to display the preprint in perpetuity. It is made available under a CC-BY 4.0 International license.

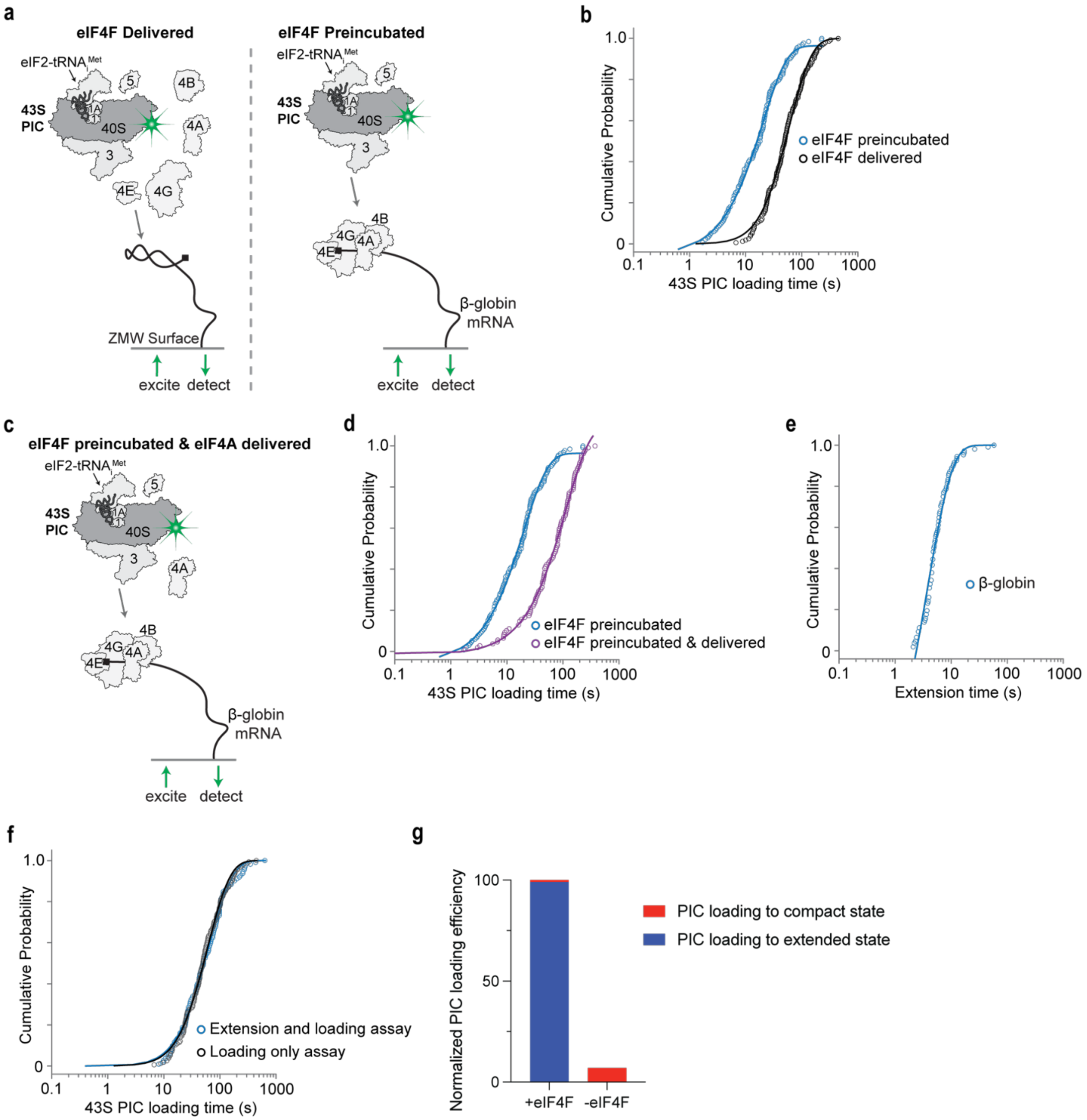

Figure S6. Extended data associated with Figure 5

a. Schematic denoting the different eIF4F conditions for the single-molecule 43S PIC loading assay. Delivered where all eIF4 proteins are delivered alongside the PIC or preincubated where the eIF4 proteins are added to the mRNA on the imaging surface prior to PIC delivery. **b.** Cumulative probability plots of 43S PIC loading time for the two conditions detailed in (**a**). Lines represent fits to either exponential (preincubated) or hypoexponential (delivered) functions; for delivered n = 156, for preincubated n = 132. **c.** Schematic showcasing the eIF4A preincubated and delivered condition for the single-molecule 43S PIC loading assay. **d.** Cumulative probability plots of 43S PIC loading time for the conditions indicated. Lines represent fits to exponential functions; for preincubated and delivered n = 109. **e.** Cumulative probability plot of extension time

bioRxiv preprint doi: https://doi.org/10.64898/2026.06.19.733470; this version posted June 19, 2026. The copyright holder for this preprint (which was not certified by peer review) is the author/funder, who has granted bioRxiv a license to display the preprint in perpetuity. It is made available under a CC-BY 4.0 International license.

from assay tracking extension and PIC loading (Fig. 5d). Lines represent fits to exponential functions, n = 129. **f.** Cumulative probability plots of PIC loading times post-synchronized to delivery of the PIC comparing two separate assays (Fig. 5a and 5d). Lines represent fits to exponential functions. **g.** Plot of normalized PIC loading efficiency (fraction of mRNA with an observed 40S binding event) with and without eIF4F, blue and red shading denote the state of the mRNA when a PIC is loaded. From left to right, n = 168 and n = 164.

bioRxiv preprint doi: https://doi.org/10.64898/2026.06.19.733470; this version posted June 19, 2026. The copyright holder for this preprint (which was not certified by peer review) is the author/funder, who has granted bioRxiv a license to display the preprint in perpetuity. It is made available under a CC-BY 4.0 International license.

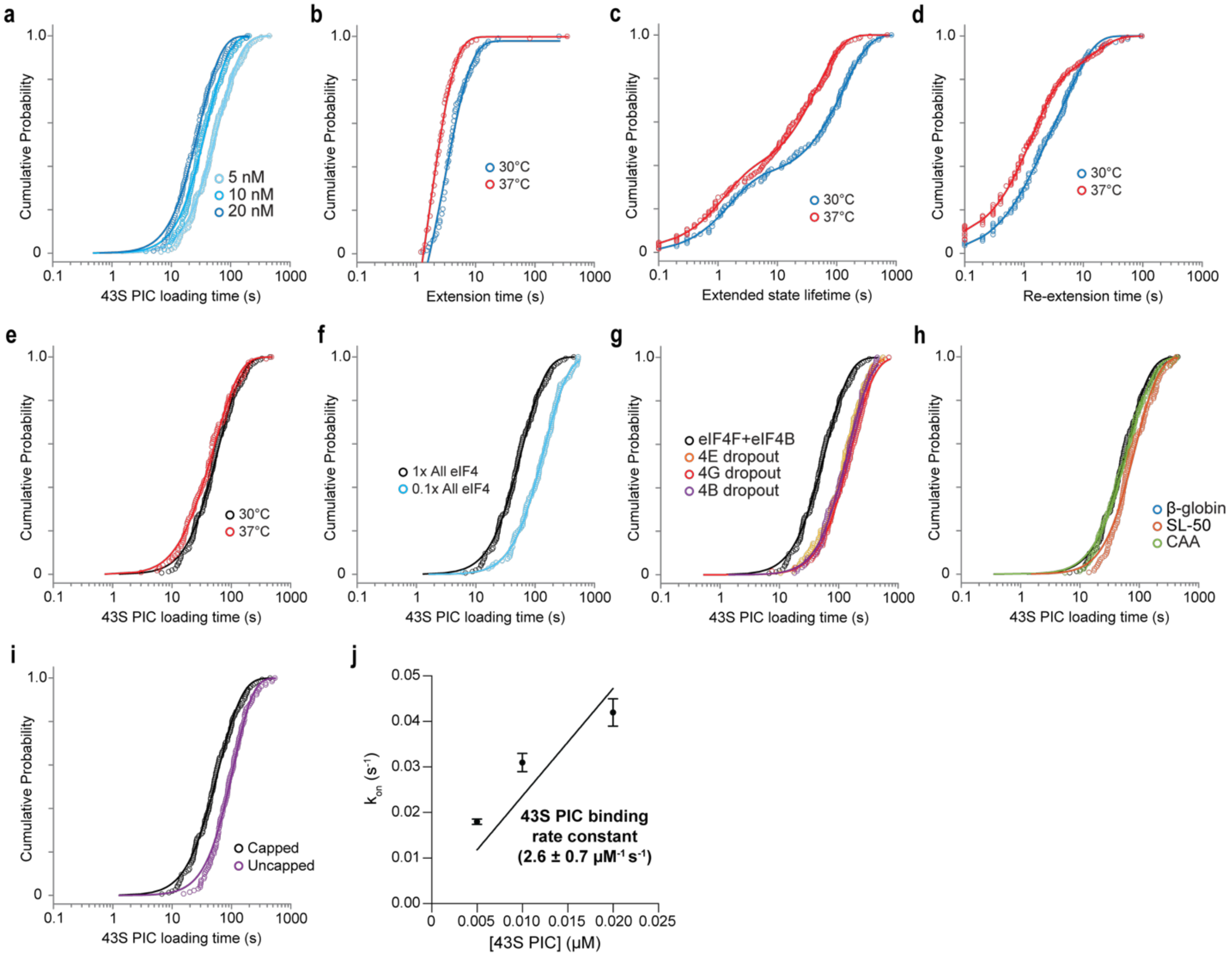

Figure S7. Cumulative probability plots associated with Figure 6

a. Cumulative probability plots of 43S PIC loading times at differing PIC concentrations. Lines represent fits to hypoexponential functions. Sample sizes displayed in Figure 6a. **b-e.** Cumulative probability plots of indicated parameters comparing two different temperatures of the reaction. Lines represent fits to either exponential (**b-d**) with n = 121 for 37 C condition, or hypoexponential functions (**e**) with n = 116 for 37 C condition. **f-i.** Cumulative probability plots of the 43S PIC loading at differing eIF4F concentrations reported by eIF4A concentration (**f**), differing eIF4 B, G, E dropouts (**g**), differing mRNAs (**h**), or differing capped states for β-globin (**i**). Lines represent fits to hypoexponential functions (**e**). In (**e**), the 0.1x eIF4 condition, n = 111. All other sample sizes were reported in Figures 6b-d. **j.** Plot of observed slow step in 43S PIC binding rate across different concentrations. Linear regression analysis (solid line) was used to derive the association rate constant. The error bars represent the 95% confidence interval (CI) of the observed association rate constants.

bioRxiv preprint doi: https://doi.org/10.64898/2026.06.19.733470; this version posted June 19, 2026. The copyright holder for this preprint (which was not certified by peer review) is the author/funder, who has granted bioRxiv a license to display the preprint in perpetuity. It is made available under a CC-BY 4.0 International license.

Table S1. Summary of 5' end-to-eIF4A FRET assay data
All rates, rate constants, mean times, number of molecules, and number of binding events analyzed in each single-molecule experiment.

Table S2. Summary of cap-to-probe FRET assay data
All rates, rate constants, mean times, and number of molecules analyzed in each single-molecule experiment. Probe positions are listed relative to the 5' cap.

Table S3. Summary of 43S PIC loading assay data
All rates, mean times, and number of molecules analyzed in each single-molecule experiment.

Table S4. Summary of FRET efficiency distribution for cap-to-probe FRET assay
Values from Gaussian fits of FRET efficiency distribution in the cap-to-probe FRET assay. Fits are for conditions with mRNA only. Mean values (μ), standard deviations (σ), normalized amplitudes (a), and 95% CI for all variables are reported.

Table S5. List of DNA templates used for mRNA *in vitro* transcription
Underlined section represents the T7 promoter sequence. Lower case sequence represents additional nucleotides remaining after linearization with XbaI.

DNA templates	Sequence (5' to 3')
β-globin	TAATACGACTCACTATAGACATTTGCTTCTGACACAACTGTGT<br>TCACTAGCAACCTCAAACAGACACCATGGTGCATCTGACTCC<br>TGAGGAGAAGTCTGCCGTTACTGCCCTGTGGGGCAAGGTGA<br>ACGTGGATGAAGTTGGTGGTGAGGCCCTGGGCAGGCTGCTG<br>GTGGTCTACCCTTGGACCCAGAGGTTCTTTGAGTCCTTTGGG<br>GATCTGTCCACTCCTGATGCTGTTATGGGCAACCCTAAGGTG<br>AAGGCTCATGGCAAGAAAGTGCTCGGTGCCTTTAGTGATGGC<br>CTGGCTCACCTGGACAACCTCAAGGGCACCTTTGCCACACTG<br>AGTGAGCTGCACTGTGACAAGCTGCACGTGGATCCTGAGAA<br>CTTCAGGCTCCTGGGCAACGTGCTGGTCTGTGTGCTGGCCC<br>ATCACTTTGGCAAAGAATTCACCCCACCAGTGCAGGCTGCCT<br>ATCAGAAAGTGGTGGCTGGTGTGGCTAATGCCCTGGCCCAC<br>AAGTATCACTAAGCTCGCTTTCTTGCTGTCCAATTTCTATTAA<br>AGGTTCCTTTGTTCCCTAAGTCCAACTACTAAACTGGGGGAT<br>ATTATGAAGGGCCTTGAGCATCTGGATTCTGCCTAATAAAAAA<br>CATTTATTTTCATTGCAAAAAAAAAAAAAAAAAAAAAAAAAAAA<br>AAAA
CAA	TAATACGACTCACTATAGCAACAACAACAACAACAACAACAAC<br>AACAACAACAACAACAACAACAACACCATGGTGCATCTGACT<br>CCTGAGGAGAAGTCTGCCGTTACTGCCCTGTGGGGCAAGGT<br>GAACGTGGATGAAGTTGGTGGTGAGGCCCTGGGCAGGCTGC<br>TGGTGGTCTACCCTTGGACCCAGAGGTTCTTTGAGTCCTTTG<br>GGGATCTGTCCACTCCTGATGCTGTTATGGGCAACCCTAAGG<br>TGAAGGCTCATGGCAAGAAAGTGCTCGGTGCCTTTAGTGATG

bioRxiv preprint doi: https://doi.org/10.64898/2026.06.19.733470; this version posted June 19, 2026. The copyright holder for this preprint (which was not certified by peer review) is the author/funder, who has granted bioRxiv a license to display the preprint in perpetuity. It is made available under a CC-BY 4.0 International license.

	GCCTGGCTCACCTGGACAACCTCAAGGGCACCTTTGCCACA CTGAGTGAGCTGCACTGTGACAAGCTGCACGTGGATCCTGA GAACTTCAGGCTCCTGGGCAACGTGCTGGTCTGTGTGCTGG CCCATCACTTTGGCAAAGAATTCACCCCACCAGTGCAGGCTG CCTATCAGAAAGTGGTGGCTGGTGTGGCTAATGCCCTGGCC CACAAGTATCACTAAGCTCGCTTTCTTGCTGTCCAATTTCTAT TAAAGGTTCCTTTGTTCCCTAAGTCCAACTACTAAACTGGGG GATATTATGAAGGGCCTTGAGCATCTGGATTCTGCCTAATAAA AAACATTTATTTTCATTGCAAAAAAAAAAAAAAAAAAAAAAAA AAAAAAAcgttggggatcctctaga
SL-50	TAATACGACTCACTATAGACAACCCTCCGGTCGACGACGGCC GATATCACGGCCGTCGTCGACCGGAGGGCACCATGGTGCAT CTGACTCCTGAGGAGAAGTCTGCCGTTACTGCCCTGTGGGG CAAGGTGAACGTGGATGAAGTTGGTGGTGAGGCCCTGGGCA GGCTGCTGGTGGTCTACCCTTGGACCCAGAGGTTCTTTGAGT CCTTTGGGGATCTGTCCACTCCTGATGCTGTTATGGGCAACC CTAAGGTGAAGGCTCATGGCAAGAAAGTGCTCGGTGCCTTTA GTGATGGCCTGGCTCACCTGGACAACCTCAAGGGCACCTTT GCCACACTGAGTGAGCTGCACTGTGACAAGCTGCACGTGGA TCCTGAGAACTTCAGGCTCCTGGGCAACGTGCTGGTCTGTGT GCTGGCCCATCACTTTGGCAAAGAATTCACCCCACCAGTGCA GGCTGCCTATCAGAAAGTGGTGGCTGGTGTGGCTAATGCCC TGGCCCACAAGTATCACTAAGCTCGCTTTCTTGCTGTCCAATT TCTATTAAAGGTTCCTTTGTTCCCTAAGTCCAACTACTAAACT GGGGGATATTATGAAGGGCCTTGAGCATCTGGATTCTGCCTA ATAAAAAACATTTATTTTCATTGCAAAAAAAAAAAAAAAAAAAA AAAAAAAAAAAAcgttggggatcctctaga

Table S6. List of DNA oligonucleotide probes used in cap-to-probe FRET assay
The notation /3'-Cy5/ represents a Cy5 dye covalently tethered to the 3' end of the oligonucleotide.

Oligonucleotides	Sequence (5′ to 3′)
Cy5-oligonucleotide probe at position +54 on β-globin (+60 on SL-50)	CCTCAGGAGTCAGATGCACC/3'-Cy5/
Cy5-oligonucleotide probe at position +73 on β-globin	GCAGTAACGGCAGACTTCTC/3'-Cy5/
Cy5-oligonucleotide probe at position +124 on β-globin (+130 on SL-50)	GCCCAGGGCCTCACCACC/3'-Cy5/
